# IL-31/33 Axis in Atopic Dermatitis

**DOI:** 10.3390/ijms262010162

**Published:** 2025-10-19

**Authors:** Julia Łacwik, Krzysztof Kraik, Julia Laska, Maciej Tota, Łukasz Sędek, Krzysztof Gomułka

**Affiliations:** 1 Student Research Group of Microbiology and Immunology, Department of Microbiology and Immunology in Zabrze, Medical University of Silesia in Katowice, 40-055 Katowice, Poland; s82960@365.sum.edu.pl (J.Ł.); s82944@365.sum.edu.pl (J.L.); 2 Student Research Group of Allergology and Internal Medicine, Clinical Department of Allergology and Internal Medicine, Wroclaw Medical University, 50-556 Wrocław, Poland; krzysztof.kraik@student.umw.edu.pl (K.K.); maciej.tota@student.umw.edu.pl (M.T.); 3 Department of Microbiology and Immunology in Zabrze, Medical University of Silesia in Katowice, 40-055 Katowice, Poland; 4 Clinical Department of Allergology and Internal Medicine, Wroclaw Medical University, 50-556 Wrocław, Poland

**Keywords:** atopic dermatitis, AD, IL-31, IL-33, IL-31/IL-33 axis, biologicaltherapy

## Abstract

Atopic dermatitis (AD) is a chronic inflammatory skin disorder characterized by impaired epidermal barrier function, immune dysregulation (e.g., Th2 polarization), genetic factors (e.g., filaggrin mutations), environmental triggers and microbial dysbiosis, leading to pruritus and eczematous lesions. In this review, we present the synergistic “IL-31/IL-33 axis.” IL-33, released by damaged keratinocytes, acts as an alarmin, initiating inflammation via ST2 receptors and promoting Th2 cytokine production (IL-4, IL-5, IL-13). This upregulates IL-31, primarily from Th2 cells, which directly activates sensory neurons to induce pruritus and impairs keratinocyte differentiation. Together, IL-31 and IL-33 exacerbate the itch–scratch feedback loop, barrier disruption, and inflammation. Elevated levels of IL-31 and IL-33 correlate with disease severity. Targeting the IL-31/IL-33 axis represents an emerging therapeutic option, e.g., nemolizumab (anti-IL-31RA) significantly reduces pruritus and AD symptoms in clinical trials. However, anti-IL-33/ST2 agents (e.g., etokimab, tozorakimab) demonstrate variable efficacy, highlighting complexity in targeting IL-33. Future research should prioritize biomarker-driven patient stratification to optimize the clinical application of these novel antibody-based therapies.

## 1. Introduction

Atopic dermatitis (AD) is a common, chronic, relapsing inflammatory skin disorder characterized by pruritus, xerosis, and eczematous lesions with variable morphology and distribution depending on the patient’s age and disease stage. It affects both children and adults, with the onset most frequently occurring in early childhood [1,2].

The prevalence of AD follows a bimodal distribution, peaking in early childhood and again in middle-aged adults, affecting about 20% of children and 5–8% of adults, with a higher incidence observed in females, along with an increasing prevalence in industrialized countries. The disease burden is significant, not only due to its symptoms but also because of its psychosocial impact and association with other atopic disorders [3,4,5,6].

It is frequently associated with physical comorbidities, including other atopic conditions such as allergic rhinitis and asthma. Indeed, AD has been increasingly recognized as a systemic condition, with established associations not only with psoriatic arthropathy but also with cardiovascular disease, diabetes mellitus, obesity, inflammatory bowel disease, and a range of hepatic comorbidities [1,7,8,9].

AD manifests with persistent daily pruritus and pain [10], often leading to significant sleep disturbances [11]. It has been demonstrated that AD significantly impairs quality of life [12]. In severe cases, these symptoms can contribute to the development of psychological complications, including depression and anxiety [13,14,15].

AD is a skin disorder resulting from a multifaceted interaction between genetic predisposition, environmental influences, immune dysregulation, and microbial imbalance. A hallmark feature of AD is an impaired epidermal barrier, frequently attributed to loss-of-function mutations in the filaggrin (FLG) gene, a structural protein essential for maintaining the stratum corneum integrity. FLG deficiency leads to increased transepidermal water loss (TEWL), enhanced permeability to allergens and irritants, and increased susceptibility to microbial colonization, notably by *Staphylococcus aureus* [16,17].

Environmental triggers, including allergens, pollutants, microbial exposure, and psychological stress, can initiate or aggravate disease activity, particularly in genetically susceptible individuals [18].

The skin microbiome plays a crucial role in the pathophysiology of AD. Patients with AD exhibit reduced microbial diversity and increased *Staphylococcus aureus* colonization on lesional and non-lesional skin. *Staphylococcus aureus* promotes inflammation through the secretion of superantigens, proteases, and toxins, which disrupt tight junctions, impair antimicrobial peptide production, and stimulate both innate and adaptive immune responses [19]. Notably, *Staphylococcus aureus* also induces IL-33 release from keratinocytes via the staphylococcal binder of immunoglobulin (Sbi), thereby disrupting barrier function and initiating downstream immune responses [20].

Immunologically, AD is characterized by a Th2-polarized T cell response. Cytokines such as IL-4, IL-5, IL-13, IL-31, and thymic stromal lymphopoietin (TSLP) promote inflammation and chronic pruritus [21]. Among these, IL-31 is a critical mediator of pruritus and eosinophilic inflammation that impairs skin barrier function. It is primarily produced by activated Th2 cells, as well as by mast cells, macrophages, dendritic cells, eosinophils, and basophils.

IL-31 exerts its biological effects through the IL-31 receptor (IL-31R), primarily expressed on non-hematopoietic tissues such as fibroblasts and endothelial cells, activating downstream signaling [22]. In parallel, IL-33 is constitutively expressed in epithelial and endothelial cells and functions both as a nuclear factor and as an extracellular cytokine (alarmin). Upon cellular damage or stress, IL-33 is released and binds to the ST2/IL-1RAP receptor complex, activating several signaling cascades, including NF-κB, JNK, and MAPK [23].

It enhances Th2 responses by stimulating IL-4, IL-5, and IL-13 production, and drives activation of eosinophils, basophils, and mast cells. Recent research has shown that IL-33 also acts directly on sensory neurons to mediate pruritus associated with dry skin, as demonstrated in human plasma studies and mouse models [24].

These two interleukins, IL-31 and IL-33, are now thought to interact synergistically in the “IL-31/IL-33 axis.” Upon exposure to allergens or pathogens, keratinocyte-derived IL-33 is cleaved into its mature form, initiating NF-κB signaling, which upregulates IL-31 gene expression. IL-4, which is itself enhanced by IL-33, further promotes IL-31 transcription [22]. Elevated serum and tissue levels of both cytokines have been correlated with disease severity and symptom intensity, further supporting the biological relevance of this axis [25].

The pathogenesis of AD is also driven by the itch-scratch cycle, a key clinical and mechanistic feature of the disease. IL-33 lowers the itch threshold through sensitization and proliferation of intraepidermal nerve fibers, while IL-31 acts directly on sensory neurons to amplify pruritic signaling. Mechanical stimuli (e.g., scratching) further disrupt the already fragile skin barrier, promoting the ingress of environmental allergens and microbes. This drives further Th2 cytokine release, particularly IL-4, IL-13, and IL-31, leading to progressive inflammation and exacerbation of itch, completing a vicious cycle [22,26,27].

In summary, the pathogenesis of AD is marked by a convergence of skin barrier defects, dysregulated Th2 immunity, and microbial dysbiosis, with the IL-31/IL-33 axis emerging as a central player in linking immune activation and neurogenic inflammation. Understanding this axis not only offers insights into disease mechanisms but also opens new avenues for targeted therapeutic interventions.

Pruritus, the most common and distressing symptom of AD, is typically accompanied by erythematous, scaly lesions that vary in morphology. The presentation and distribution of AD symptoms depend largely on the patient’s age. In infants, AD usually appears within the first year of life as erythematous papules, patches, or plaques on the face (particularly the cheeks), scalp, trunk, and extensor surfaces of the limbs, such as the outer arms and legs, while the diaper area is usually spared. Older children often develop lesions on flexural areas, while adults commonly exhibit dry, scaly patches primarily on the extremities. At this stage, chronic scratching often leads to lichenification and excoriation. In adolescents and adults, the condition frequently persists or re-emerges in the same flexural regions but may also involve the hands, upper arms, face-particularly the eyelids-and the neck. Chronic lesions in this age group are often characterized by lichenification, scaling, and post-inflammatory pigmentary changes [2,28].

Current treatment strategies for AD primarily focus on restoring skin barrier function through the use of emollients, alongside the avoidance of exacerbating factors and adjunctive therapies such as topical corticosteroids and topical proactive therapy. Commonly employed topical treatments include corticosteroids and calcineurin inhibitors, including tacrolimus. Additionally, oral antihistamines and anti-allergic drugs may be used to alleviate symptoms. In more severe cases, systemic therapies such as oral corticosteroids, cyclosporine, and phototherapy with narrow-band ultraviolet B (Nb-UVB) are implemented. Recent advances in understanding the immunological basis of AD have facilitated the development of targeted biologic therapies, offering new options for patients with refractory disease. These include agents such as dupilumab, which inhibits IL-4 and IL-13 pathways, and omalizumab, an anti-IgE monoclonal antibody, which are increasingly utilized in moderate-to-severe cases [5,29,30,31].

This review synthesizes recent findings on IL-31 and IL-33 in AD, identifies gaps and conflicting data in current knowledge, and proposes research directions to elucidate the mechanistic interplay between these cytokines in AD pathogenesis and inflammation. Additionally, the review explores modern biological interventions targeting IL-31 and IL-33 and their efficacy, safety profiles, and therapeutic potential in AD management.

## 2. Materials and Methods

### 2.1. Search Strategy

A literature search was conducted using the PubMed, Embase, and Cochrane Library databases. The following keywords were used in the search strategy: ““atopic dermatitis” AND (IL-33 OR IL-31 OR anti-IL-31 OR anti-IL-33 OR IL31 OR IL33)”. The term used in the Embase search engine was “(‘atopic dermatitis’/exp OR ‘atopic dermatitis’) AND (‘interleukin 33’/exp OR ‘interleukin 33’ OR ‘interleukin 31’/exp OR ‘interleukin 31’)”.

### 2.2. Inclusion and Exclusion Criteria

Studies were eligible for inclusion in this review based on the following criteria: (1) article published in a peer-reviewed journal, (2) article written in the English language, (3) human or animal studies, and (4) study related to the review’s subject.

Exclusion criteria included: (1) publication types such as conference proceedings, book chapters, letters to editors, and case reports, (2) abstracts without full-text available, (3) articles published in a language other than English, (4) articles published in non-peer-reviewed journals, and (5) studies deemed not related to the current review’s subject.

### 2.3. Literature Selection

**Figure 1:**
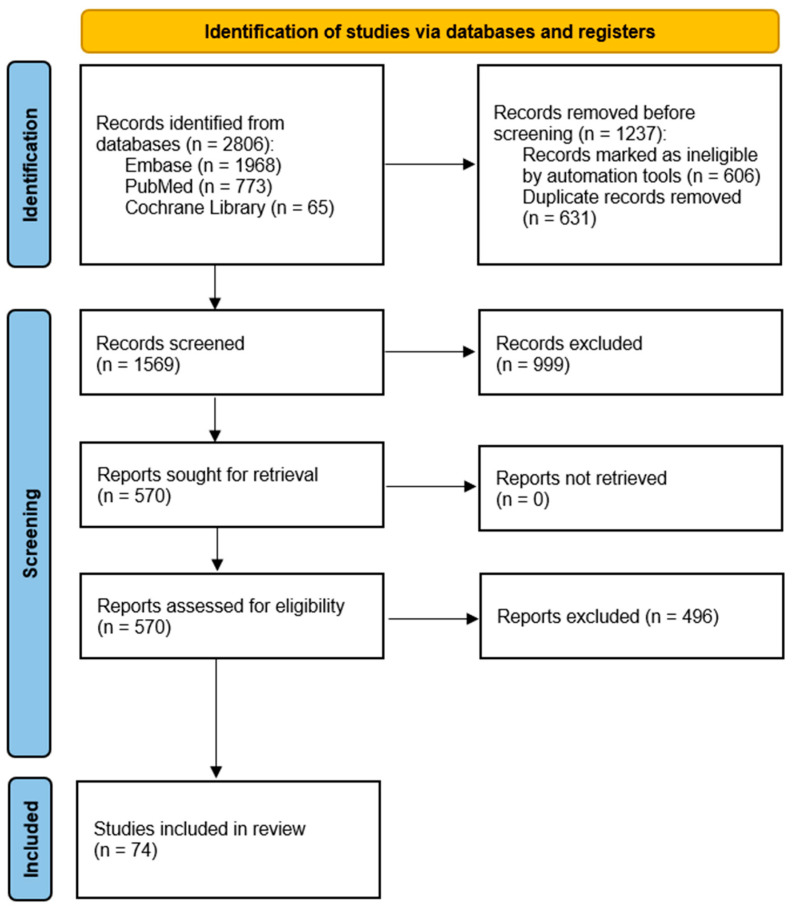
PRISMA flow diagram presenting study selection process.

Four independent reviewers assessed studies published before 10 September 2025. During the screening process, priority was given to recent evidence, particularly studies published since 2020. Articles were first assessed based on their titles and abstracts, and if found relevant, the full texts of the studies were retrieved. Subsequently, methodological quality and scientific validity were evaluated. Finally, all articles were required to fulfill the predefined inclusion and exclusion criteria (as detailed in the respective subsection) to be incorporated into the review. The selection process is presented in the form of a flow diagram in Figure 1.

## 3. IL-31 in Atopic Dermatitis

**Figure 2:**
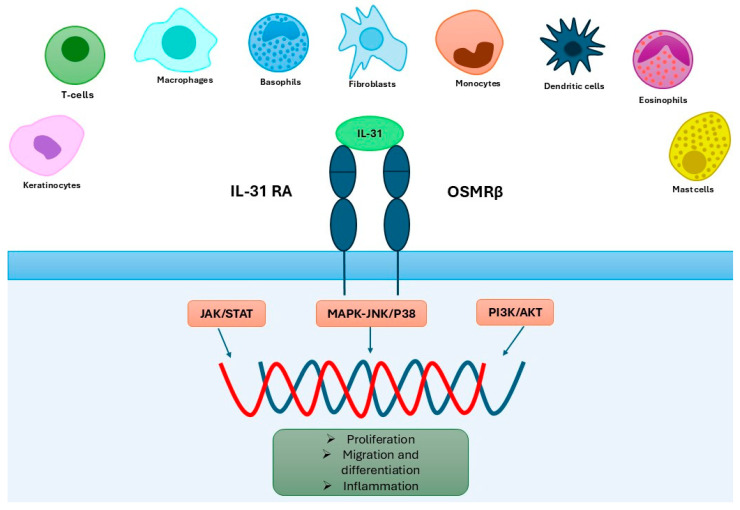
Key cell types involved in IL-31 signaling and the three associated intracellular signaling pathways.

IL-31 is a 4-helix cytokine from the IL-6 family, known for its pro-inflammatory properties and a shared signaling pathway engaging in gp130 receptor subunit activation [32,33]. IL-31 mRNA and protein expression are largely restricted to CD4^+^ T cells, particularly activated T helper 2 (Th2) cells and skin-homing CD45RO^+^ cutaneous lymphocyte-associated antigen-positive (CLA^+^) memory T cells. Increased numbers of CLA^+^CD45RO^+^ T cells in AD skin suggest their contribution to IL-31 production and disease pathogenesis [34,35]. Besides Th2 cells, IL-31 is also secreted, though less abundantly, by Th1, CD8^+^ T cells, monocytes, dendritic cells, keratinocytes, fibroblasts, basophils, mast cells, and eosinophils [35,36,37,38,39]. IL-31 signals through a heterodimeric receptor consisting of the gp130-like subunit IL-31 receptor alpha (IL-31RA) and oncostatin M receptor beta (OSMRβ). Through the receptor complex, IL-31 induces the activation of JAK/STAT (Janus-activated kinase/signal transducer and activator of transcription), PI3K/AKT (phosphatidylinositol 3-kinase/protein kinase), and MAPK-JNK/p38 (mitogen-activated protein kinase-Janus kinase/p38) pathways [40]. IL-31RA is naturally expressed by undifferentiated keratinocytes, eosinophils, and small sensory neurons located in the dorsal root and trigeminal ganglia, particularly in the dermal branches of sensory nerve fibers [41]. OSMRβ mRNA is broadly expressed in many tissues [42] [Figure 2].

Pruritus is a hallmark and most burdensome symptom of AD, resulting from complex pathomechanisms, including epidermal barrier dysfunction, type 2 inflammation, and neural sensitization. Damaged skin facilitates allergen penetration, leading to the activation of Th2 immune responses and the secretion of cytokines, including IL-4, IL-13, and IL-31. These mediators contribute to sensory neuron activation, intraepidermal nerve fiber elongation, and amplification of the pruritus-scratch cycle [43].

IL-31 contributes to the severity of pruritus in mammals [44,45]. It also promotes epidermal proliferation, contributing to skin remodeling and thickening during the chronic phase of the disease [46]. IL-31 plays a role in the pathophysiology of AD, acting on both keratinocytes and sensory neurons. In epidermal keratinocytes, IL-31 binds to the IL-31RA/OSMRβ receptor complex, leading to an increased expression of pro-inflammatory chemokines (e.g., CCL17, CCL22, CXCL1) and suppression of differentiation markers such as filaggrin, involucrin, and cytokeratin 10. It also induces IL-20 and IL-24, suggesting a role in regulating differentiation. Since filaggrin loss is linked to AD, IL-31 may contribute to its pathogenesis. At the same time, IL-31 increases antimicrobial peptides (AMPs) such as S100A7, S100A8, S100A9, and β-defensins via IL-1α. High IL-31 levels impair keratinocyte differentiation, whereas low levels promote AMP expression—indicating a dual role in barrier disruption and immune defense [47,48,49]. IL-31 also stimulates the surface expression of intercellular adhesion molecule-1 (ICAM-1) on eosinophils and fibroblasts, enhancing leukocyte adhesion and tissue infiltration [46]. IL-31 is also found in eccrine sweat, where it can stimulate keratinocytes to produce the pro-inflammatory cytokine CCL2, further contributing to local skin inflammation [50]. As mentioned above, *Staphylococcus aureus*–derived superantigens may significantly contribute to the upregulation of IL-31 expression. These exotoxins exacerbate inflammation by upregulating IL-31RA expression on human monocytes and macrophages, thereby enhancing IL-31-induced production of pro-inflammatory cytokines and AMPs in both macrophages and keratinocytes [46,51]. A recent study demonstrated that although canine atopic skin exhibits reduced expression of key barrier proteins, keratinocytes from atopic and healthy dogs showed no intrinsic differences when reconstructed in vitro. Barrier disruption was only reproduced following exposure to pro-inflammatory cytokines. Among these, IL-31, TNF-α, IL-4, and IL-13 contributed to filaggrin downregulation and structural defects. These findings indicate that IL-31 may function as a direct promoter of epidermal barrier dysfunction [52].

In the nervous system, IL-31 directly stimulates small-diameter sensory neurons, particularly C-fibers, which co-express IL-31RA and OSMRβ. This interaction promotes neurite growth and increases the density of intraepidermal nerve fibers, enhancing neuronal sensitivity and chronic pruritus. These effects are mediated by STAT3 signaling and suggest a key role for the IL-31-nerve axis in the sensory dysregulation observed in AD [53,54]. Notably, human dorsal root ganglion neurons, many of which co-express the transient receptor potential cation channel subfamily V member 1 (TRPV1) receptor, also express IL-31RA. In vivo studies show that blocking TRPV1 disrupts IL-31 signaling, highlighting TRPV1 as a potential therapeutic target in pruritus [54].

Numerous studies report elevated serum IL-31 levels in patients with AD compared to healthy subjects, regardless of the disease subtype and age of the patients [55,56,57,58,59,60,61]. IL-31 levels remained elevated not only during periods of exacerbation, but also during remission, suggesting persistent inflammatory activity of a subclinical nature [57]. In addition, in children with AD, IL-31 levels correlated with markers of eosinophilic inflammation [58]. A significant correlation between IL-31 levels and disease severity markers, such as Scoring Atopic Dermatitis (SCORAD) index, and subjectively assessed pruritus intensity has been repeatedly demonstrated [58,59,61]. However, several studies have reported no significant correlation between serum IL-31 levels and clinical measures of disease severity or pruritus intensity in AD patients, suggesting that the relationship between IL-31 and clinical manifestations may be more complex and possibly influenced by additional factors [55,56,60].

## 4. IL-33 in Atopic Dermatitis

**Figure 3:**
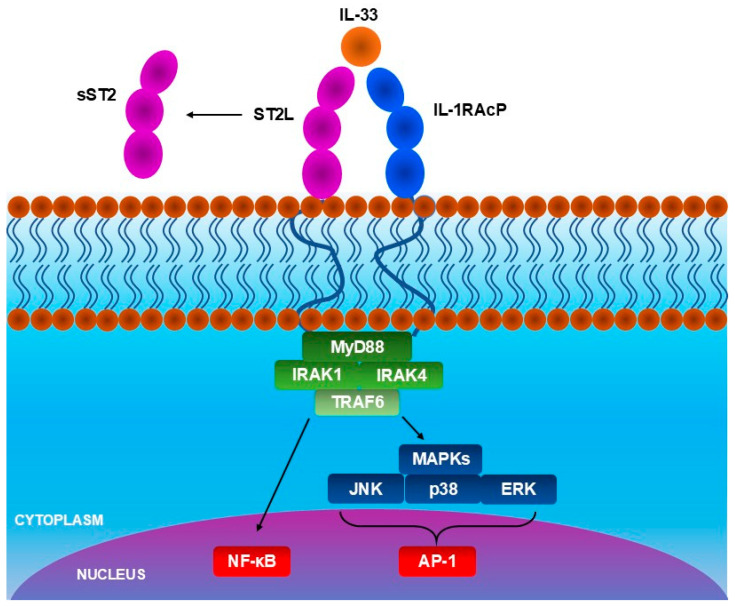
The IL-33/ST2 pathway. IL-33 binding to membrane ST2 activates intracellular signaling pathways, transducing IL-33 bioactivity. Soluble ST2 (sST2) functions as a decoy receptor, sequestering IL-33 and inhibiting signaling.

Interleukin-33 (IL-33) is a cytokine that plays an important role in inflammatory and allergic diseases, including AD [62,63,64,65]. It is constitutively produced, most importantly, in epithelial and endothelial cells, which are barrier cells, as well as in fibroblast-like cells, myofibroblasts, and in other cells, including oligodendrocytes and astrocytes, gastrointestinal and urogenital smooth muscle cells, and CD45^+^ cells. Prior to its release, IL-33 is localized in the cellular nucleus, bound to H2A and H2B histones [66], presumably regulating gene expression [67]. IL-33 is an alarmin and is released when barrier cells are damaged, making its release an alarm signal [65]. The types of damage triggering IL-33 release include mechanical injury, scratching, bacterial, fungal, or viral infections, contact with allergens, and induction of necrosis. During apoptosis, caspase-3 and caspase-7, enzymes that cleave IL-33, are activated, which reduces the activity of IL-33 upon release [68]. Consequently, IL-33 activity after apoptosis is low. Secondary to the damage, the level of IL-33 is elevated temporarily [62]. However, within a few hours, it returns to a normal level. It was previously confirmed that mechanical stress causes the secretion of IL-33. However, there is no strong evidence confirming whether cell death is required to occur to release this cytokine. Other factors considered potential causes of IL-33 secretion include extracellular ATP, uric acid, and thapsigargin. IL-33 belongs to the IL-1 family and participates in allergic inflammation, type-1 and type-2 immunity. Increased level of IL-33 was observed in asthma, chronic obstructive pulmonary disease (COPD), and colitis. The activity of IL-33 can be modified by enzymes, including proteases released by immune cells and mast cell chymase, which increase IL-33 activity [64]. IL-33 activates cells with expression of interleukin 1 receptor-like 1 (IL1RL1), also known as ST2 receptor, being a member of the toll-like/IL-1-receptor superfamily [65], and IL-1 receptor accessory proteins (IL1RAcP) co-receptor: T helper 2 (Th2) cells, T regulatory cells (Treg), mast cells, group 2 innate lymphoid cells (ILC2s), eosinophils [69], and other immune system cells [62]. ST2 receptors are also localized on dermal fibroblasts [69] and epithelial cells [70]. It was found that the expression of both ST2 and IL-1RAcP mRNA is increased in lesional skin of AD patients when compared to non-lesional skin in human subjects [71]. In the same study, ST2 and IL-33 expression also increased in the murine AD model after, respectively, 3 weeks and 1 week of allergen sensitization. There are two types of ST2 receptors: membrane-bound ST2 receptors, which activate cells, and soluble ST2 receptors, which are decoy receptors and neutralize IL-33 [64,72]. Interaction between IL-33 and membrane-bound ST-2 causes the activation of the myeloid differentiation-primary response protein 88 (MyD88), which further activates the IL-1R-associated kinase 1 (IRAK1), IRAK4, and TNFR-associated factor 6 (TRAF6), leading to transcription of nuclear factor-ĸB (NF-ĸB) and mitogen-activated protein kinases (MAPK), JAK2/STAT3, PI3K/Akt/mTOR, and CaMKII/CREB pathways (Figure 3). The activation of these kinases leads to the production and release of pro-inflammatory mediators, including IL-31, proliferation and activation of mast cells, and exacerbation of allergic diseases.

The IL-33/ST2 pathway was found to play a role in pain and pruritus development in chronic diseases, including AD, inflammatory diseases, neuropathic pain, and cancer [72]. Patients with AD have increased IL-33 levels in both skin and blood. It was found that increased serum level of IL-33 may be caused by skin mechanical damage or barrier disruption, e.g., due to scratching, and exacerbate skin inflammation in AD [73]. Tamagawa-Mineoka et al. investigated the differences in IL-33 serum levels among subjects with AD, psoriasis, urticaria, and healthy individuals. The serum level of IL-33 was significantly higher in AD patients in comparison to other groups. The IL-33 level correlated with the severity of excoriation and xerosis, but not with the severity of pruritus, erythema, lichenification, edema, crust, and serum IgE or eosinophil levels. The level of IL-33 was reduced after effective treatment of AD. The authors of the cited study suggested that IL-33 may have potential use in monitoring the process of AD treatment. Moreover, in a murine model of allergic contact dermatitis, blocking IL-33/ST2 signaling, either IL-33 or ST2 with an appropriate antibody, alleviated both inflammation and pruritus. This treatment also significantly reduced skin bifold thickness and TEWL and dermatitis score following daily antibody administration. Conversely, administration of exogenous IL-33 exacerbated the pruritus, which was histamine-independent and ST2-dependent [74].

In contrast to these pre-clinical findings, phase 2 randomized clinical trial of astegolimab, a human IgG antibody that binds to IL-33 receptor, did not demonstrate a significant improvement in AD symptoms [75]. In an editorial commentary on this clinical trial, Schuler and Gudjonsson suggest that the role of IL-33 in AD may be more critical in the initiation phase of inflammation rather than in sustaining chronic, ongoing inflammation [76].

**Figure 4:**
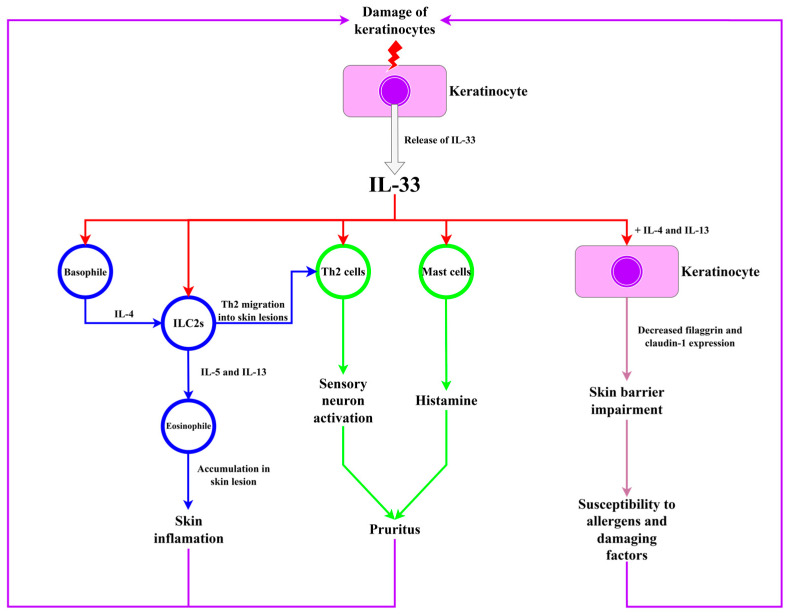
The role of IL-33 in AD, based on Imai’s study [77].

Currently available studies suggest that IL-33 plays an important role in inflammation and skin barrier disruption in AD (Figure 4) [77]. After its release from damaged keratinocytes, located in skin lesions, IL-33 activates ILC2s responsible for innate immunity, which cause Th2 cells, which are responsible for acquired immunity, to migrate into lesions. ILC2s activation causes reversible dermatitis, while Th2 activation causes irreversible changes. IL-33 stimulates the activation of type 2 immune response through activation of previously mentioned ILC2s, Th2 cells, and other cell types, including mast cells and basophils. The activation of these cells induces the production and release of type 2 immune response cytokines, including IL-4, IL-5, IL-13, and IL-31, and histamine. In the chronic phase of AD, apart from previously mentioned cell types, Th1, Th17, and Th22 cells contribute to further progression of the lesions. Imai, in his review, distinguished the following mechanisms that take part in IL-33-dependent skin lesion development:

1. IL-33 activates basophils and ILC2s through ST2 receptor, and IL-4 released by basophils further increases the activity of ILC2s. ILC2s produce more IL-5 and IL-13, causing the accumulation of eosinophiles in skin lesions.
2. IL-33 activates Th2 cells, inducing the release of IL-31, which directly activates sensory neurons, causing pruritus. Moreover, IL-33 activates mast cells, which release histamine—another agent responsible for pruritus. In response to the pruritus, scratching occurs, which causes further damage to keratinocytes and the release of IL-33 again, exacerbating the inflammation.
3. IL-33 and cytokines which are releases due to IL-33-dependent immune cell activation—IL-4 and IL-13, reduce filaggrin expression impairing the skin barrier. Moreover, IL-33 activates the signal transducer and activator of transcription 3 (STAT3) pathway, reducing expression of claudin-1. Skin with disrupted barrier function is more susceptible to allergens and other harmful factors, leading to damage to keratinocytes and an increase in IL-33 release.

Savinko et al. investigated the role of IL-33 in AD in humans and a murine model [71]. AD patients exhibited significantly increased ST2, IL1RAcP, TNFα, and mRNA expression of IL-13 in lesional skin compared to non-lesional skin. Furthermore, the increase in IL-33 was also present, but it did not reach statistical significance. No difference in expression of these factors was revealed between the non-lesional skin of AD patients and the skin of healthy subjects. Moreover, house dust mites in AD patients with house dust mite allergy and patients exposed to staphylococcal enterotoxin B had an increased expression of ST2 and IL-33 mRNA in the skin over time. In the murine AD model, sensitization with ovalbumin caused an increase in expression of ST2, IL-33, Th2-type cytokines (IL-4 and IL-13), and the number of mast cells and eosinophils in the skin. ST2, IL-33, IL-4, and IL-13 mRNA, TNFα, and IFNγ were also upregulated in mice after staphylococcal enterotoxin B exposure. Also, in filaggrin-deficient mice, the basal expression of ST2 and IL-33 was higher. Furthermore, topical treatment with tacrolimus decreased the increased expression of IL-33 and ST2 in mice, but corticosteroid treatment did not affect the expression of either IL-33 or ST2.

Furthermore, Borek et al. investigated the role of IL-33 in AD in a canine model and found that IL-33 is significantly overexpressed in the chronic phase of AD [78]. The study included 49 dogs divided into subgroups based on the type of lesions, including lichenification, eczema, and non-lesional pruritus, and 10 of these dogs were healthy and assigned to a control group. IL-33 immunoreactivity in keratinocytes was significantly higher in the lichenification group when compared to other groups. Moreover, the correlations were found between IL-33 immunoreactivity and age, Canine Atopic Dermatitis Extent and Severity Index-4 (CADESI-4) score, and epidermal thickness. The study suggests that IL-33 has a significant role in the development of AD and skin inflammation in the chronic phase of AD. Moreover, IL-33 may contribute to the development of acanthosis. However, the effect of IL-33 on pruritus seems indirect and may be mediated by Th2 cells activation and secretion of IL-31.

Another study was conducted by Seltmann et al. [79]. The keratinocytes and fibroblasts of AD patients and healthy people were isolated and stimulated with cytokines, including TNFα, IFNγ, and IL-1β. It was found that keratinocytes isolated from patients with AD have higher membrane-bound ST2 receptor expression than healthy subjects and that its expression is unaffected by stimulation with cytokines used in the study. Moreover, the expression of IL-33 mRNA and IL-33 protein in keratinocytes was upregulated by IFNγ. The upregulating effect of IFNγ was stronger in AD patients than in healthy participants of the study. However, stimulation with TNFα reduced the overexpression of IL-33 protein induced by IFNγ. Furthermore, IL-33 stimulated CD4^+^ T cells to release more IFNγ, creating a positive feedback loop. Moreover, TNFα and IL-1β stimulation increased the IL-33 mRNA expression in skin-resident fibroblasts. The stimulation with both TNFα and IL-1β had a greater effect than stimulation with TNFα or IL-1β alone. Moreover, Savinka et al. found that stimulation with both TNFα and IFNγ increases the expression of IL-33 mRNA in human dermal fibroblasts, HaCaT keratinocytes, macrophages, and HUVEC endothelial cells [71]. Stimulation of fibroblasts using IFNγ only had a small effect on IL-33 expression, while the stimulation with TNFα or IL-4, or both of them at once, did not affect the IL-33 expression in either keratinocytes or fibroblasts.

On the contrary, a study conducted by Xuan et al. found that circulating IL-33 level was negatively associated with the risk of AD [80]. Another study, authored by Zaryczańska et al., included 191 AD patients and 168 healthy people, and also found no difference in serum level of IL-33 between AD patients and healthy subjects, and no association was found between serum IL-33 level and pruritus intensity, IgE level, or SCORAD index, which is used to assess the severity of AD [81]. Also, no significant association was found between the frequency of polymorphisms in the -9894 T/C (rs1929992) and -11877 C/T (rs10975519) IL-33 loci between AD patients and healthy subjects. Only the TT genotype in the 11,877 locus increased the risk of pruritus occurrence, and it was more common in patients experiencing severe and very severe pruritus.

A recent study by Gunji et al. elucidated a novel role of IL-33 in AD pathogenesis [82], demonstrating its function in priming the nucleotide-binding oligomerization domain-like receptor family pyrin domain containing 3 (NLRP3) inflammasome in basophils. Using murine bone marrow-derived basophils, the authors showed that IL-33 increased the expression of NLRP3 and IL-1β at the mRNA level, and NLRP3 and pro-IL-1β at the protein level. This effect was confirmed in vivo, where IL-33 administration increased IL-1β expression in basophils, but not eosinophils, in the lungs of mice. Mechanistic studies using pathway inhibitors revealed that IL-33 mediates this upregulation of NLRP3 and IL-1β via the NF-κB and p38 MAPK pathways, whereas JNK and ERK1/2 pathways were not involved. Furthermore, the NF-κB pathway was shown to regulate the expression of IκBζ, which also contributes to NLRP3 inflammasome priming; subsequent IL-1β release was found to occur through gasdermin D pores. In a murine AD model, the study demonstrated that IL-1β in AD lesions is derived from basophils, and that basophil depletion abolished IL-1β production and neutrophil recruitment. This pathway was further confirmed using NLRP3 knockout mice. In summary, IL-33 activates the NF-κB and p38 MAPK pathways in basophils, upregulating pro-IL-1β and NLRP3 expression. These components, alongside apoptosis-associated speck-like protein containing a caspase recruitment domain (ASC), and caspase-1, assemble into the NLRP3 inflammasome. Active caspase-1 then cleaves pro-IL-1β into its mature form and processes gasdermin D to form membrane pores, facilitating the release of IL-1β and inducing pyroptosis [82].

In a separate study, Chen et al. revealed a role for IL-33 in keloid formation [83]. Analysis of human keloid tissue showed a significant increase in IL-33 expression in the epidermis, but not the dermis, of both peripheral and interior keloid zones and non-lesional skin, compared to mature scars. ST2 receptor expression was significantly elevated in the superficial dermis of keloids, with CD45^+^ lymphocytes and myelocytes, particularly CD4^+^ T cells, identified as the primary ST2-expressing cells. In vitro experiments on HaCaT keratinocytes demonstrated that IL-33 release from keratinocytes stimulated lymphocytes to produce IFN-γ, which in turn activated the JAK1/STAT1 pathway in keratinocytes, as confirmed by JAK1 inhibition, leading to further IL-33 production and establishing a positive feedback loop. Additionally, IFN-γ stimulation and the consequent increase in IL-33 levels disrupted HaCaT keratinocyte differentiation, as evidenced by decreased expression of filaggrin and involucrin. The specific role of IL-33 was confirmed by the finding that IL-33 knockdown restored normal filaggrin and involucrin levels, suggesting that this cytokine drives the skin barrier dysfunction and chronic inflammation observed in keloids. Although not directly examined in their study, the authors note that similar changes have been documented in AD keratinocytes, implying a potential analogous role for IL-33 in impairing keratinocyte differentiation in AD [83].

In another recent study [84]. Guo et al. identified hypomethylation and overexpression of multiple genes, including SEMA7A, in AD children. Furthermore, stimulation of HaCaT keratinocytes with SEMA7A protein increased the expression of both IL-33 and IL1RL1 mRNA in these cells. Authors of the cited study suggested that SEMA7A may become a therapeutic target in AD treatment in the future.

## 5. The IL-31/IL-33 Axis

The IL-31/IL-33 axis is a pathway that includes the interplay between IL-33 and IL-31 signaling, in which IL-33 enhances IL-31 signaling and vice versa. This axis plays an important role in several diseases, including AD, allergic contact dermatitis, psoriasis, asthma, allergic rhinitis, viral infections, e.g., bronchiolitis in the course of rhinovirus infection, neoplastic diseases, osteoporosis, and autoimmune diseases, including systemic lupus erythematosus, systemic sclerosis, and rheumatoid arthritis [65,85,86,87,88]. In these diseases, a simultaneous increase in both IL-33 and IL-31 was observed, and their levels correlated with the severity of the diseases’ symptoms. Moreover, the IL-31/IL-33 axis might have a role in intracerebral hemorrhage. However, this statement needs to be examined further, as high IL-31 serum level was associated with higher National Institutes of Health Stroke Scale (NIHSS) score, hemorrhage volume, and poor prognosis [89], while a high serum level of IL-33 was a marker of good prognosis, and it was negatively correlated with baseline NIHSS score and hemorrhage volume [90]. This discrepancy suggests that IL-33 and IL-33 have different roles in intracerebral hemorrhage than in previously mentioned diseases.

**Figure 5:**
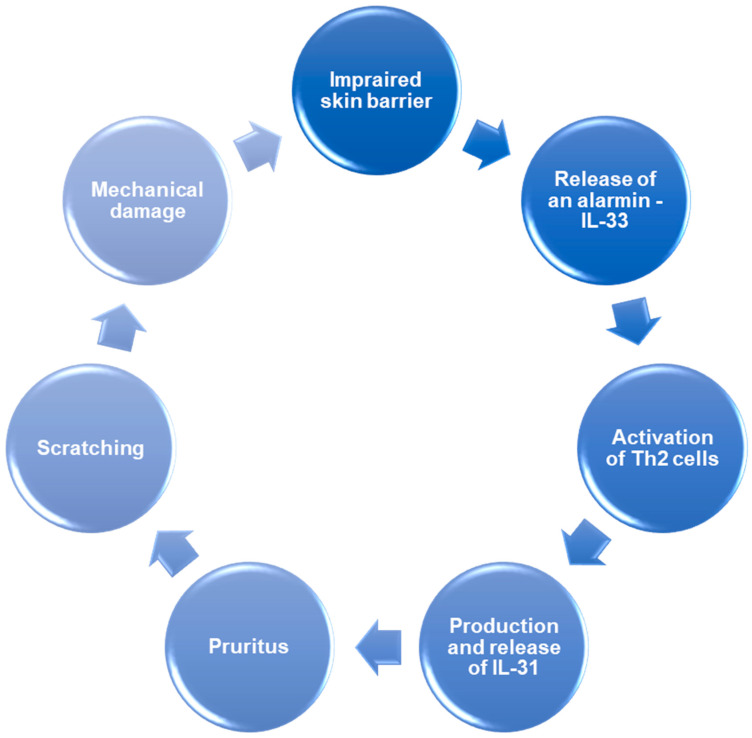
The amplification loop of the IL-31/IL-33 axis in the course of AD.

In the mechanistic basis of the IL-33/IL-31 axis, IL-33 initiates inflammation, while IL-31 enhances neurons’ sensitivity, causing pruritus [87]. As previously mentioned, IL-33 is an alarmin that is released when barrier cells are damaged and die [62]. The activation of the IL-33/ST2 pathway causes the activation of Th2 cells and mast cells, which are the producers of IL-31. Successively, IL-31 exacerbates inflammation and causes pruritus by directly stimulating sensory neurons and enhancing their sensitivity [54,65,87], resulting in scratching [91], which causes further dermal cell damage and release of IL-33 [73]. Moreover, IL-31 induces epidermal cell proliferation and thickening of the epidermis, further disrupting the barrier function of the skin, making it more susceptible to damage [65]. An amplification loop is created connecting the roles of IL-33 and IL-31 in the form of a vicious circle of inflammation, pruritus, and scratching (Figure 5). Currently, the research is ongoing to develop new drugs that could influence this amplification loop, especially the IL-33/ST2 activity, and disrupt this positive feedback [65]. The IL-31/IL-33 axis was successfully affected in a study conducted by Che et al. [92]. In this study, luteolin was successfully used to reduce IL-31 mRNA and protein expression, as well as IL-31 release, in human mast cells induced by IL-33 in cell culture. However, it should also be noted that some other drugs, including epidermal growth factor receptor tyrosine kinase (EGFR-TK) inhibitors, may activate the IL-31/IL-33 axis as their adverse effect and initiate dermatitis, presumably by damaging keratinocytes and causing the release of IL-33 [93].

The p53 family member p63 (ΔNp63) is a regulator of transcription of both IL-33 and IL-31 in keratinocytes. ΔNp63 overexpression was observed in lesional skin samples of AD patients, in comparison to non-lesional skin of AD patients and skin of healthy people [94]. In the mouse model, the increase in ΔNp63 expression induced the development of pruritus and lesions similar to AD in areas accessible for scratching, including erythema, erosions, and lichenification. The severity of dermatitis symptoms was increasing over time. The epidermis of mice with ΔNp63 overexpression was thicker when compared to mice with normal ΔNp63 expression. Moreover, the acanthosis, hyperkeratosis, parakeratosis, and hypogranulosis were observed. Furthermore, the serum level of IgE was elevated, while the expression of epidermal differentiation markers was reduced at the level of both mRNA and protein. In the epidermis of mice with increased ΔNp63 expression, numerous Th2 signaling-related cytokines and chemokines were overexpressed, especially IL-33. The genes of IL-31 and its receptors (IL31RA and OSMR) were also overexpressed, suggesting the role of ΔNp63 in IL-33 and IL-31 signaling. After the suppression of ΔNp63 overexpression, the skin symptoms and lesions have regressed, and IL-33 and IL-31 signaling, as well as IgE levels, have been normalized. Another molecule that likely affects the IL-31/IL-33 axis is intelectin, which causes overexpression of IL-33 [95].

Furthermore, it was found that *Staphylococcus aureus* skin colonization can also increase the constitutive release of IL-33 from human keratinocytes in cell culture. This process, mediated by *Staphylococcus aureus* immunoglobulin-binding protein as identified through fractionation and candidate testing, promotes a type 2 immune response [20]. The release of IL-33 occurs independently of Toll-like receptor signaling. The same study demonstrated that in a murine model, IL-33 was essential for initiating the immune response against *Staphylococcus aureus*; notably, neither *Staphylococcus epidermidis* nor group A *Streptococci* induced this type 2 response.

In the murine AD model, consisting of filaggrin-deficient mice, Staphylococcus enterotoxin B increased the expression of IL-33 2-fold and ST2 4.5-fold after 24 h [71]. Following topical tacrolimus administration, IL-33 and ST2 expression was reduced 2-fold and 4-fold, respectively, whereas treatment with betamethasone-17-valerate was ineffective. Additionally, multiple cell lines, including human dermal fibroblasts, human HaCaT keratinocytes, HUVEC endothelial cells, and human primary macrophages, produced IL-33 in response to both TNF-α and INF-γ. A mimetic of double-stranded RNA increased IL-33 production in fibroblasts, suggesting that both bacterial and viral infections may upregulate IL-33 production in skin cells [71].

Moreover, exposure to *Staphylococcus* α-toxin and *Staphylococcus* enterotoxin B significantly increased the expression of IL-31RA at the mRNA (quantified by quantitative RT-PCR) and protein levels (assessed by flow cytometry), in peripheral blood mononuclear cells (PBMCs) and macrophages isolated from humans [96]. Stimulation of PBMCs and macrophages with IL-31 and staphylococcal toxins led to changes in the release of pro-inflammatory cytokines, respectively, IL-6 and IL-1β from PBMCs, and IL-6, IL-1β, IL-8, and IL-18 from macrophages. Findings from these studies support the role of infections, especially *Staphylococcus aureus* colonization, in the activation of the IL-31/IL-33 axis [96].

In conclusion, due to the IL-31/IL-33 axis, there exists a positive feedback loop that amplifies the skin damage and symptoms of AD. Collectively, these findings underscore the role of microbial infections, particularly colonization with *Staphylococcus aureus*, in activating the IL-31/IL-33 axis. The expression and signaling of both IL-33 and IL-31 can be further modified by various factors, including ΔNp63 or intelectin. The elements of this loop may become targets for new therapeutic methods used in AD in the future.

## 6. Therapeutic Targeting of IL-31/IL-33 Axis

**Figure 6:**
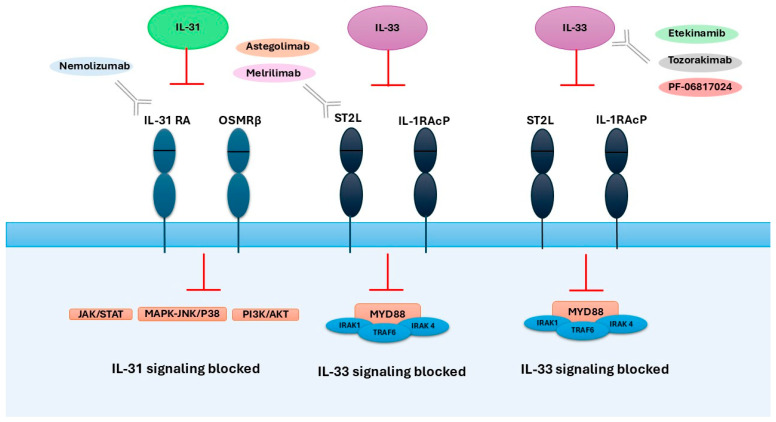
Molecular targets of biological therapies acting on the IL-31 and IL-33 axis in atopic dermatitis.

Modulation of the IL-31/IL-33 axis may constitute a promising therapeutic strategy for selected patients with AD [Figure 6]. Clinical manifestations such as pruritus can be effectively controlled through the use of monoclonal antibodies targeting specific mediators within this cytokine pathway.

### 6.1. Therapies Targeting IL-31

Nemolizumab is a humanized monoclonal antibody against IL-31RA that inhibits IL-31 signaling and suppresses pruritus by competitively preventing IL-31 from binding to IL-31RA [97,98]. It demonstrates great efficacy in reducing symptoms of AD in many phase II and phase III studies in recent years [98,99,100,101,102,103].

A randomized, double-blind, 16-week phase 3 study evaluated the efficacy and safety of subcutaneous nemolizumab (60 mg every 4 weeks) in Japanese patients with AD and moderate to severe pruritus who had an inadequate response to topical treatment. Patients were randomly assigned in a 2:1 ratio to receive nemolizumab or placebo, with continuation of topical treatment. The primary endpoint was the mean percent change in Visual Analogue Scale (VAS) for pruritus from baseline to week 16. Nemolizumab significantly reduced pruritus compared to the placebo and also led to greater improvements in the Eczema Area and Severity Index (EASI), quality of life (QoL), and sleep. Injection site reactions were reported in 8% of patients receiving nemolizumab versus 3% with placebo [104].

Long-term efficacy and safety were assessed in two phase 3 trials (JP01 and JP02) involving patients ≥13 years with AD and persistent moderate-to-severe pruritus. Both studies administered 60 mg of nemolizumab subcutaneously every 4 weeks with topical therapy. In JP01, patients received nemolizumab or placebo for 16 weeks, followed by 52 weeks of open-label treatment. JP02 provided 52 weeks of active treatment from the outset. An 8-week follow-up phase followed each study. Sustained reductions in pruritus (66% VAS decrease) and skin symptoms (78% EASI decrease) were observed by week 68. QoL improvements appeared after the first dose and persisted throughout. No serious adverse effects (AEs) were reported [105].

The ARCADIA 1 and ARCADIA 2 phase 3 trials included adolescents and adults (≥12 years) with moderate-to-severe AD inadequately controlled with topical corticosteroids. Participants received nemolizumab (30 mg every 4 weeks after a loading dose of 60 mg) or placebo, with topical therapy. After 16 weeks, a significantly higher proportion of patients in the nemolizumab group achieved success per the Investigator Global Assessment (IGA). Improvements were also observed in all key secondary endpoints, including pruritus reduction, sleep quality, and overall clinical response. Therapeutic effects were noticed since the first week. The safety profile was similar between groups, with no severe treatment-related events or deaths [106].

In a pediatric phase 3 trial, children aged 6–12 years with moderate-to-severe AD and pruritus unresponsive to topical treatment and antihistamines received 30 mg nemolizumab or placebo subcutaneously every 4 weeks for 16 weeks, with concurrent topical treatment. The primary endpoint was the change in weekly pruritus score (5-point scale). Nemolizumab significantly reduced pruritus severity vs. placebo, with effects visible as early as day 2. Secondary endpoints, including AD symptoms and QoL, also favored nemolizumab. No treatment discontinuations due to adverse events occurred [107].

The most common adverse events related to nemolizumab were skin-related. These included AD exacerbation (10–30%), new-onset eczema, erythema, urticaria, acne, contact dermatitis, and skin infections (including fungal). Patients were accompanied by pruritus, pain, and xerosis, as well as a burning sensation. In some cases, psoriasis-like reactions and bullous pemphigoid were observed, potentially due to immune shifts (Th2/Th17 overactivation) after IL-31 blockade. Most reactions were mild and responsive to topical treatment; treatment discontinuation remained below 10% [108]. Additional symptoms included impaired sleep, emotional distress, and reduced daily functioning [109]. As an example, although the drug is intended to alleviate sleep disturbances associated with itching and improve daytime functioning, it paradoxically caused marked hypersomnolence after each injection in a 16-year-old boy with severe AD. Although skin symptoms and itching improved significantly, the patient repeatedly experienced excessive daytime sleepiness, which only resolved after discontinuation of treatment. Possible causes include changes in circadian rhythm or effects on the central nervous system associated with IL-31 inhibition, although the exact mechanism has not yet been established, so it is important to carefully monitor both dermatological outcomes and daytime alertness [110].

A case of asthma exacerbation associated with nemolizumab administration has also been reported [111]. Side effects in long-term studies were mild and similar to those reported in prior studies [105,112,113]. A recent retrospective study investigated clinical predictors of nemolizumab efficacy in AD. Among 14 Japanese patients, longer disease duration was significantly associated with a poorer treatment response, while baseline serum IgE levels and EASI scores alone showed only moderate predictive accuracy. Notably, combining these parameters substantially improved the ability to distinguish between responders and nonresponders, with certain combinations (e.g., disease duration with IgE or EASI) correctly classifying up to 100% of patients. These results suggest that disease duration, serum IgE levels, and baseline EASI scores may serve as valuable predictors of clinical outcomes with nemolizumab therapy in AD [114].

In the analysis by the National Institute for Health and Care Excellence (NICE), the list price of nemolizumab is £2257 per 30 mg vial, although the actual costs to the NHS remain lower due to a confidential discount agreement. Official, available sources do not provide an annualized cost calculation for nemolizumab therapy [115]. As an illustration, for dupilumab, an American economic analysis indicates a list price of approximately $37,000 per year (300 mg every two weeks), a figure which fell within the range of its value-based price—defined as the annual maintenance therapy price at which a drug is cost-effective—calculated at $28,769–$39,941 per year [116]. The NICE report emphasized that in comparative analyses, nemolizumab was not cost-effective compared to JAK inhibitors, as their actual net prices (covered by confidential agreements in the NHS) are lower and thus provide a more favorable cost-effectiveness profile. Consequently, although nemolizumab was considered acceptable in comparison with other biologics, its clinical availability may be limited by competition from JAK inhibitors, which represent a more cost-effective alternatives [115].

### 6.2. Therapies Targeting IL-33

Etokimab (ANB020), a humanized IgG1 antibody directed against IL-33, was evaluated in a phase 2a study in 12 adult patients with moderate to severe AD. After a single intravenous dose of 300 mg, rapid and marked clinical benefit was observed, with 83% of patients achieving EASI-50 by day 29 and 33% achieving EASI-75, with sustained improvement through day 57. Treatment significantly reduced peripheral blood eosinophil counts (by ~40% on average) and reduced neutrophil infiltration in the skin after allergen (house dust mite) provocation. In ex vivo studies, etokimab inhibited IL-33-induced neutrophil migration and secondarily reduced their sensitivity to IL-8 by inhibiting CXCR1 receptor expression, confirming its immunomodulatory effect on the IL-33-CXCR1 axis. In addition, patients demonstrated improvements in disease assessment measures, including SCORAD, DLQI, and the 5-D pruritus scale. Etokimab was well tolerated, and AEs (e.g., headaches, respiratory infections) were mild and transient. These results indicate the important role of IL-33 in the pathogenesis of AD and the therapeutic potential of its blockade [117].

Tozorakimab (MEDI3506), another anti–IL-33 antibody, was studied in a phase 2a trial (FRONTIER-2; NCT04212169) involving 148 adults with moderate-to-severe AD. Patients received either placebo or tozorakimab (60, 300, or 600 mg) subcutaneously every 4 weeks for 16 weeks. The primary endpoint (EASI score change at week 16) did not reach statistical significance in any group. However, numerically higher EASI-75 and IGA 0/1 response rates were seen in the 600 mg group. The drug showed dose-proportional pharmacokinetics, low immunogenicity, and good tolerability. While the study did not achieve statistical significance in its primary outcome, the observed numerical improvements in key clinical endpoints suggest potential therapeutic activity of tozorakimab in AD, warranting further investigation in larger trials [118]. In the FRONTIER-2 trial, the overall incidence of treatment-emergent adverse events (TEAEs) and serious adverse events was comparable between tozorakimab and placebo groups, with no heart failure–related events reported. Injection site reactions were more frequent with tozorakimab, constituting the most notable treatment-related adverse effect [119].

Astegolimab (MSTT1041A) is a fully human immunoglobulin G2 monoclonal antibody that selectively inhibits the IL-33 receptor, ST2 [120]. In a Phase 2 randomized, placebo-controlled study, adult patients with chronic AD received either astegolimab 490 mg or placebo, administered subcutaneously every four weeks for 16 weeks. The primary objective was to evaluate improvement in disease severity using the EASI score. A total of 65 patients were enrolled. Both the astegolimab and placebo groups showed reductions in EASI scores at week 16, but no significant difference was observed between the two groups. Secondary outcomes and biomarker analyses were also comparable. Astegolimab was well-tolerated, with no severe AEs. Pharmacokinetic data confirmed adequate drug exposure from the first week of treatment [75].

The phase 1 study evaluated the safety, tolerability, pharmacokinetics, pharmacodynamics, and immunogenicity of a monoclonal antibody, melrilimab (CNTO 7160), directed against the IL-33R [121,122]. The study included 15 patients with mild AD who received three intravenous doses of CNTO 7160 at two-week intervals (3 mg/kg or 10 mg/kg) or placebo. The drug was well-tolerated; the only serious AE was cellulitis in the 3 mg/kg group. After three intravenous doses (3 or 10 mg/kg every 2 weeks), rapid and sustained inhibition of free sIL-33R and strong dose-dependent inhibition of p38 phosphorylation in basophils were observed. Despite the confirmed biological effect, no improvement in clinical parameters (SCORAD, EASI) was observed, most likely due to the benign nature of the disease and the short duration of treatment. Nevertheless, the data obtained suggest that melrilimab effectively inhibits the IL-33 pathway and may represent a potential therapeutic option in AD, requiring further research on a broader patient cohort. [122].

PF-06817024 is a humanized monoclonal antibody with high affinity for IL-33. In preclinical studies, PF-06817024 demonstrated the ability to effectively bind and neutralize IL-33, thereby inhibiting activation of the IL-33/ST2 pathway and reducing the production of pro-inflammatory cytokines. A phase 1 trial in moderate-to-severe AD patients involved a saturating intravenous dose (600 mg), followed by 3 doses of 300 mg every 4 weeks. PF-06817024 was generally well tolerated, and AEs were mild to moderate. Participants with AD reported skin-related symptoms, such as increased eczema or pruritus, among others, but their frequency was comparable between the study and placebo groups. Significant pharmacodynamic effects of the drug were confirmed by observing dose-dependent increases in total serum IL-33 levels, indicating effective binding and blocking of the molecular target. The pharmacokinetics of the drug showed a long half-life (approximately 90 days), which may allow for less frequent dosing in future studies. The results support the further development of PF-06817024 as a potential therapy for patients with AD [123].

Itepekimab, an IgG4P anti–IL-33 antibody, was evaluated in two prematurely terminated phase 2 trials. One study assessed monotherapy with various doses; the second compared itepekimab alone and combined with dupilumab, a human monoclonal IgG4 antibody directed against IL-4 and IL-13. Despite good tolerability and lack of anti-drug antibodies, itepekimab did not significantly improve AD symptoms (EASI or other measures). Combination with dupilumab also failed to enhance efficacy beyond that of dupilumab alone, leading to early study termination [124,125].

Analysis of the failures of IL-33 blockade studies reveals both common and drug-specific factors. All studies confirmed the pharmacodynamic effect, including target saturation and reduction in inflammation biomarkers; however, the lack of translation into significant clinical improvement suggests that the role of IL-33 in the pathogenesis of AD is context-dependent and limited to specific disease endotypes.

In the case of itepekimab, the premature study termination was due to lack of efficacy compared to placebo and the lack of additional benefits from combination therapy with dupilumab, thereby undermining the hypothesis of an additive effect [124]. For astegolimab, despite adequate exposure and reduction in eosinophils clinical outcomes were not improved, and the lack of consistent biomarker trends suggests a marginal effect of ST2 blockade in modulating chronic inflammation [75]. Although tozorakimab successfully bound its target in serum, it may not have achieved effective concentrations in the skin. Furthermore, the results were further confounded by a high placebo response and the inclusion of patients with milder disease [118]. Similarly, despite confirmed binding to the IL-33 receptor and potent inhibition of p38 phosphorylation in basophils, CNTO 7160 also failed to demonstrate a clinical effect [122].

Taken together, these results indicate that isolated IL-33 blockade, while effective at signal inhibition, does not provide a therapeutic effect in heterogeneous populations [Table 1]. Contributing factors include compensation by alternative inflammatory axes, limited drug penetration into target tissues, and a lack of patient stratification by biomarker profiles render the potential benefits invisible in clinical trials.

## 7. Current Challenges and Future Directions

Emollients and proactive topical therapy remain underutilized in clinical practice despite well-established evidence of their efficacy. Proactive therapy that includes twice-weekly application of corticosteroids or calcineurin inhibitors (e.g., tacrolimus) to previously affected areas following lesion resolution, prevents AD exacerbations by maintaining subclinical inflammation control. This strategy requires concurrent daily emollient use to optimize skin barrier repair. The limited implementation of these approaches in standard clinical practice underscores a persistent gap between research evidence and its practical application. Enhancing awareness of and adherence to proactive management protocols, particularly among primary care providers and patients, remains a significant challenge.

In more severe cases, systemic treatments and novel antibody-based therapies are introduced. Therapeutic targeting of the IL-31/IL-33 axis represents a novel biologic strategy for AD management. In this review, we emphasize the complexity of inhibiting the IL-31/IL-33 axis, which demonstrates consistent evidence of target engagement yet yields variable clinical outcomes. Anti-IL-31 receptor α (IL-31RA) agents (e.g., nemolizumab) demonstrate significant reductions in pruritus and disease severity across clinical trials. In contrast, IL-33/ST2 inhibitors (e.g., etokimab, tozorakimab) exhibit heterogeneous clinical responses, underscoring the pathobiological challenges of IL-33 modulation. Although recent clinical data appear promising, several challenges remain. For nemolizumab, consistent efficacy is evident across diverse patient populations; however, predicting individual responses and managing immune-related adverse effects, such as paradoxical skin reactions, remain debatable. For certain anti-IL-33 therapies, variability in clinical outcomes despite confirmed biological activity suggests that the role of IL-33 in AD may be context-dependent and potentially restricted to specific disease endotypes. These findings emphasize the need for biomarker-driven patient stratification in future clinical trials, including markers related to IL-31 and IL-33, to better identify subgroups that are most likely to benefit from IL-31/IL-33 axis inhibition.

Despite consistent evidence that both IL-31 and IL-33 are involved in the pathogenesis of AD, their exact role remains unclear. Discrepancies in clinical trial results for IL-33, conflicting associations between serum IL-33 levels and disease severity, and the lack of robust biomarkers point to a significant knowledge gap. In particular, it is unclear which AD endotypes are primarily driven by the IL-31/IL-33 axis and why IL-31 inhibition provides consistent results while IL-33 blockade does not.

This review, therefore, aims to assess the IL-31/IL-33 axis in atopic dermatitis, highlight existing uncertainties, and emphasize the need to develop biomarker and endotype-based therapeutic strategies targeting this pathway.

This systematic review was conducted in accordance with PRISMA guidelines. However, due to the absence of consistent, quantifiable endpoints across the included studies, a meta-analysis was not feasible. The findings are therefore presented as a qualitative synthesis. In line with the objectives of a qualitative synthesis, a formal risk-of-bias assessment using tools was not implemented.

Future research should prioritize the identification of a broader range of modulators within the IL-31/IL-33 axis to identify additional therapeutic targets for drug development. Simultaneously, integrating clinical, molecular, and immunological data is essential to refine disease endotyping and optimize therapeutic strategies. Reliable biomarkers reflecting IL-31/IL-33 axis activation could be critical for guiding personalized treatments, enhancing therapeutic efficacy, and minimizing adverse effects. Furthermore, exploring combination therapies and novel axis modulators may provide new therapeutic avenues for the effective management of treatment-resistant AD.

## Figures and Tables

**Table 1:** Drugs targeting the IL-31/33 axis in atopic dermatitis. AD—atopic dermatitis; IV—intravenous; EASI—Eczema Area and Severity Index, QoL—quality of life (QoL), SCORAD—Scoring Atopic Dermatitis; IGA—Investigator Global Assessment.

Drug	Mechanism of Action	Dose	Response to Treatment	Adverse Effects	Ref.
Nemolizumab	Humanized monoclonal antibody against IL-31RA	60 mg every 4 weeks (30 mg in children)	Reduction in puritis and AD symptoms; significant improvements in quality of life.	Mild; mainly skin-related adverse events; deterioration in the quality of sleep, emotional sphere, and daily activities.	[104,105,106,107,108,109]
Etekinamib	Humanized IgG1 anti-IL-33 monoclonal antibody	Single IV 300 mg dose	Reduction in AD symptoms and skin inflammation; sustained clinical improvement up to day 57; significant improvement in QoL and pruritus.	Mostly mild and transient; common: headache, upper respiratory infections; no serious drug-related safety concerns	[117]
Tozorakimab (MEDI3506)	Human monoclonal antibody that neutralizes IL-33	60 mg, 300 mg, or 600 mg subcutaneously every 4 weeks (×4 doses)	No statistically significant difference in primary endpoint (EASI score at Week 16); numerical increase in EASI-75 and IGA 0/1 responses in the 600 mg group.	Mostly mild/moderate; 2 discontinuations (600 mg); 1 serious case of thrombosis (300 mg) linked to treatment.	[118,126]
Astegolimab	Human IgG2 monoclonal antibody that blocks IL-33 receptor ST2	490 mg subcutaneously every 4 weeks for 16 weeks	No significant difference vs. placebo in EASI score improvement at Week 16; similar secondary outcomes and biomarkers.	Mostly mild/moderate AEs; common: worsening AD, infections; few related AEs; 1 serious unrelated AE; no withdrawals due to AEs.	[75]
Melrilimab (CNTO 7160)	Monoclonal antibody targeting the IL-33R	3 mg/kg or 10 mg/kg IV every 2 weeks (3 doses total)	Strong, dose-dependent inhibition of p38 phosphorylation in basophils and sIL-33R neutralization; no clinical improvement (SCORAD, EASI).	Well tolerated; one serious adverse event (cellulitis) in the 3 mg/kg group; moderate immunogenicity; no deaths reported	[122]
PF-06817024	Humanized monoclonal antibody that binds and neutralizes IL-33	Loading dose: 600 mg IV, then 300 mg IV every 4 weeks (total 4 doses)	Dose-dependent increase in total IL-33 indicating target engagement; exploratory efficacy data (e.g., clinical symptom improvement) to be published separately.	Mostly mild to moderate; included AD exacerbation (15%), eczema (10%), nausea (10%), and infections (20%). One treatment-related serious AE was reported (cervical carcinoma)	[123]
Itepekimab	Human monoclonal IgG4P antibody that binds IL-33	SC: 300 mg every 2 weeks, 300 mg every 4 weeks, 100 mg every 4 weeks, 30 mg every 8 weeks	No clinically significant improvement in EASI scores compared to placebo; no added benefit in combination with dupilumab.	Well tolerated; most common: nasopharyngitis, worsening of AD.	[124]

## Data Availability

Data sharing is not applicable as no datasets were generated or analyzed during the current study.
